# Climate Influence on Deep Sea Populations

**DOI:** 10.1371/journal.pone.0001431

**Published:** 2008-01-16

**Authors:** Joan B. Company, Pere Puig, Francesc Sardà, Albert Palanques, Mikel Latasa, Renate Scharek

**Affiliations:** 1 Institut de Ciències del Mar-Consejo Superior de Investigaciones Científicas (ICM-CSIC), Barcelona, Spain; 2 Centro Oceanográfico de Gijón, Instituto Español de Oceanografía (IEO), Gijón, Spain; University of Sheffield, United Kingdom

## Abstract

Dynamics of biological processes on the deep-sea floor are traditionally thought to be controlled by vertical sinking of particles from the euphotic zone at a seasonal scale. However, little is known about the influence of lateral particle transport from continental margins to deep-sea ecosystems. To address this question, we report here how the formation of dense shelf waters and their subsequent downslope cascade, a climate induced phenomenon, affects the population of the deep-sea shrimp *Aristeus antennatus*. We found evidence that strong currents associated with intense cascading events correlates with the disappearance of this species from its fishing grounds, producing a temporary fishery collapse. Despite this initial negative effect, landings increase between 3 and 5 years after these major events, preceded by an increase of juveniles. The transport of particulate organic matter associated with cascading appears to enhance the recruitment of this deep-sea living resource, apparently mitigating the general trend of overexploitation. Because cascade of dense water from continental shelves is a global phenomenon, we anticipate that its influence on deep-sea ecosystems and fisheries worldwide should be larger than previously thought.

## Introduction

Vertical, often seasonal, sinking of organic material is widely accepted as the main source of nutrients to the deep-sea floor [1]–[3]. The life histories of animals dwelling in the vast expanses of the deep-sea realm mainly rely on particles sinking from the euphotic layers of the open ocean where primary production is generally low [4]–[6]. Despite this scenario of low productivity, the deep-sea sustains surprisingly large biomasses of predatory fish [7]–[9]. However, the increase of deep-sea fisheries [10]–[12] has often lead to a depletion of these stocks after only a few years of exploitation [10], [13]. Among the overexploited stocks worldwide [14], [15], Mediterranean fisheries are considered to be particularly susceptible to overexploitation [16]. Surprisingly, Mediterranean stocks have not collapsed [17]. One of the most striking example of this paradox is the Mediterranean deep-sea shrimp *Aristeus antennatus* (Risso, 1816), a target species of a monospecific fishery, which has been intensively trawled in the deep-sea for more than 70 years.

Settlement and preferential recruitment areas for this species occurs well below 1000 m [18], [19]. Postlarval individuals (<10 mm CL; carapace length) have been only found at 1200 m depth during winter-time, and juveniles <20 mm CL are only present at non-fishing depths below 1000 m depth [20], being animals under age 2 [21]–[23]. Small juveniles of this species dwell at depths below 1000 m and undertake ontogenetic migrations to shallower grounds (500–900 m) where fishing takes place, mainly on adult populations [24] (see Figure S1 for details). Several models have been published regarding the long-term fluctuations of *Aristeus antennatus* landings (population dynamic, bioeconomical and production models) and, up to date, none of them have provided the biological or environmental causes of the interannual landing fluctuations (see [25]–[28] among other references). The fact that *Aristeus antennatus* is one of the most valuable fishing resources of the Mediterranean allowed us to use an extensive official data base to conduct this study. However, the official-origin sources only indicate catch data (kilograms per year/month/day) depending on the source of information separated in two sales categories (large and small individuals), without differentiating between males and females (see Material and Methods). No historical time-series data on individual size frequencies were available which would have allowed building a consistent modeling approach.

**Figure 1:**
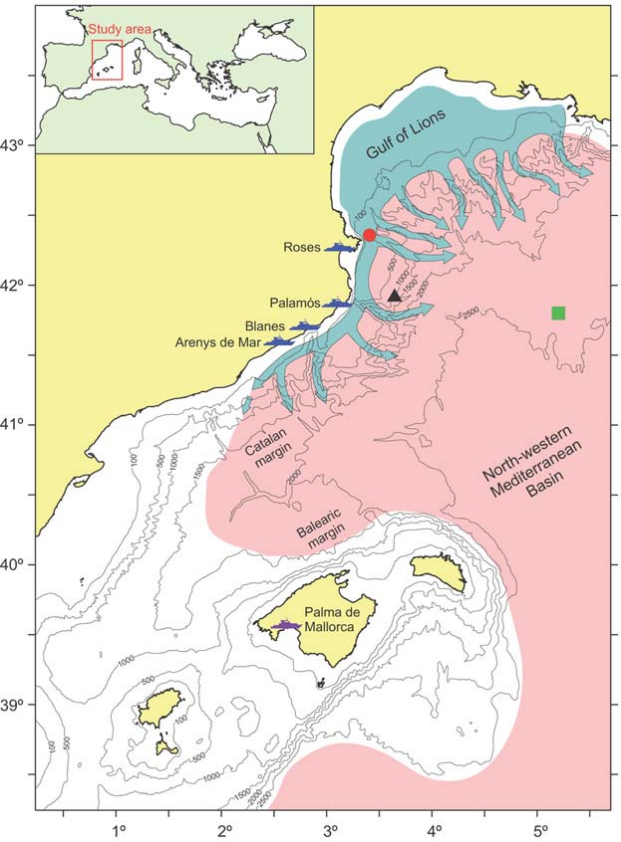
Map of the study area. Bathymetric map of the northwestern Mediterranean showing the location of the fishing harbors considered in this study (blue ships). Landings from Palma de Mallorca fishing harbor (purple ship) are included as supporting information (Figure S5). Moorings in the Cap de Creus Canyon (red circle) and in the basin (green square), and the Wavescan buoy deployed at 1200 m water depth off Palamós (black triangle) are also shown. Pale blue arrows indicate the pathway of the dense shelf water cascading mechanism extending from the Gulf of Lions along and across the continental slope, and the faded pink area represents the region affected by the thermo-haline and turbidity anomaly observed in the Western Mediterranean Deep Water after the 1999 and 2005 major cascading events. Both phenomena are inferred from published data [27]–[33].

The northwestern Mediterranean is one of the regions of the world where massive open ocean dense water formation occurs because of wind-induced cooling and evaporation of surface waters during winter-time [29]. Concurrent with this phenomenon, coastal surface waters over the wide shelf of the Gulf of Lions also become denser than the underlying waters and cascade downslope, mainly through submarine canyons, until reaching their equilibrium depth [30], [31]. In very dry, windy and cold winters, such as in 2005, cascading is exceptionally intense [32]. Under these circumstances, dense shelf waters propagate along and across the continental slope [33], [34], reaching depths >2000 m, generating a thermo-haline and turbidity anomaly in the Western Mediterranean Deep Water that spreads over the entire northwestern Mediterranean basin [32], [35], [36] (Figure 1). Previous intense cascading events of dense shelf waters (i.e. those reaching the deep basin) were identified after the analysis of historical hydrographic data and occurred in 1971, 1980, 1988 and 1999 [37], therefore occurring at a decadal time-scale.

In this study, we present a mechanism of interaction across ecosystems by describing how a climate-driven phenomenon originated in shelf environments and occurring at a decadal time-scale influences the ecology of a deep-sea population, apparently mitigating fishery overexploitation. We first provide evidence that the physical disturbance originated by strong currents during intense cascading events co-occur with the disappearance of the individuals of a deep-sea living resource from the fishing grounds. Second, we show that the temporal evolution of annual landings of this species is correlated in time and space by the occurrence of intense cascading events. We finally demonstrate that an increase in landings of this species is explained by an enhancement of its recruitment process probably favored by the large transport of particulate organic matter to the basin during intense cascading events.

## Results and Discussion

**Figure 2:**
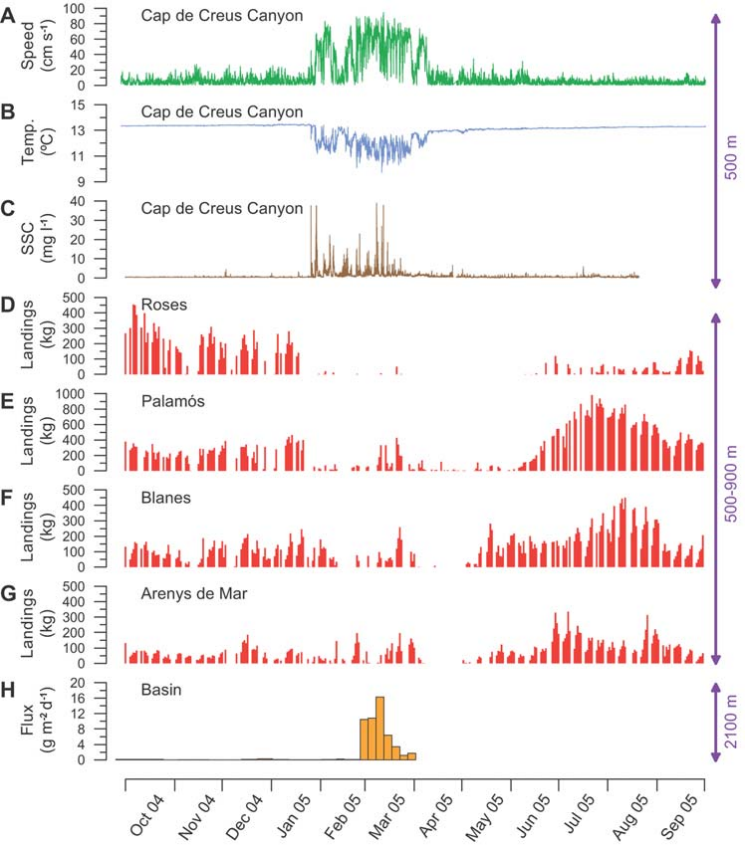
Mooring time series and daily catches. (A–C) Current speed (A), temperature (B) and suspended sediment concentration (SSC) (C), recorded in the Cap de Creus Canyon at 500 m depth (placed at 5 m above bottom), before, during and after the cascading event of winter 2005. (D–G) Daily landings of *Aristeus antennatus* at the studied harbors are plotted as red bar charts, ordered from northeast (Roses) to southwest (Arenys de Mar). (H) Downward mass fluxes recorded at 2100 m depth by the sediment trap moored in the basin at 2350 m depth (i.e. 250 m above bottom) are illustrated by the orange bar chart. See locations in Figure 1.

**Figure 3:**
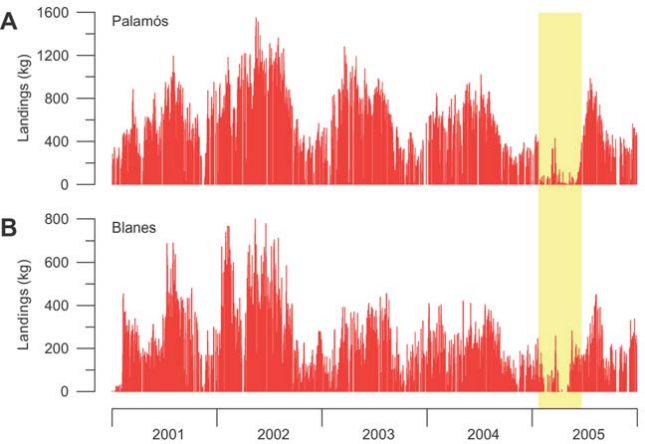
Intra-annual variability of daily catches. (A and B) Daily landings of *Aristeus antennatus* since 2001 at Palamós (A) and Blanes (B), the most representative studied harbors. Captures show a marked pattern of intra-annual variability with higher landings in spring-summer and lower landings in autumn, which has been attributed to reproductive displacements of the population [19]. The pale yellow band shows the period affected by the temporary fishery collapse caused by the winter 2005 dense shelf water cascading event, disrupting the intra-annual variability of precedent years.

During the 2005 cascading event, downslope currents associated with the propagation of dense, cold and turbid shelf waters reached speeds >85 cm s^−1^ inside submarine canyons of the northwestern Mediterranean (Figure 2A–2C). The analysis of the daily landings of *Aristeus antennatus* in the studied harbors (see Figure 1 for locations) reveals that the potential dragging capacity of such energetic flows with high loads of particles in suspension coincide in time and space with the disappearance of this deep-sea living resource from the fishing grounds. Dense shelf water cascading in 2005 co-occurred with a temporary fishery collapse of this shrimp that lasted 1–5 months, depending on the harbors (Figure 2D–2G), disrupting the intra-annual variability of its landings (Figure 3A and 3B). This temporary fishery collapse was not related to unusual wave conditions that could have kept the fleets in port during winter 2005 (Figure S2) neither was it related to a reduction of the number of trawlers during this period [38], as they are regulated by law. Therefore the potential fishing effort through this time interval was approximately the same. However, it is true that the disappearance of *Aristeus antennatus* from the fishing grounds leads to a temporary reduction of the real fishing effort of this monospecific fishery, but only as long as this species does not return to these grounds. It is worth to note that from late January to early June 2005, i.e. during the temporary fishery collapse period, daily landings in Palamós and Blanes harbors were ∼50–100 kg (Figure 2E and 2F), indicating that few trawlers were still operating and acting as “spotters”. As soon as those boats bring large catches to the fish markets, the rest of the specialized fleet returns to the deep-sea fishing grounds.

Cascading currents in the Cap de Creus Canyon were detected at 500 m depth in late January 2005, coinciding with the disappearance of this shrimp from the landings of the closest harbors (i.e. Roses and Palamós). This disappearance was gradually delayed in the harbors located down current and towards the southwest (i.e. Blanes and Arenys de Mar), in agreement with propagation of the cascading event [33], [34]. Landings at Roses and Palamós progressively recovered 2–3 months after cessation of the cascading observed in the Cap de Creus Canyon, indicating that effects on this deep-sea population can last for longer than the event itself (Figure 2D–2G). The same disappearance pattern of *Aristeus antennatus* from the fishing grounds occurred in 1999, during the previous major cascading event (Figure S3A and S3B). The spatio-temporal co-occurrence between cascading and the temporary fishery collapse of *Aristeus antennatus* suggest that the physical disturbance by such strong deep currents of cold and turbid water probably displaces the individuals of this species from the fishing grounds, presumably towards greater depths.

Our observations in the northwestern Mediterranean basin during winter 2005 show that particle fluxes at 2100 m depth (250 m above the bottom ) increased abruptly by more than 2 orders of magnitude (Figure 2H), because of the lateral particle transport associated with the arrival of dense shelf waters to the basing after the intense cascading event [34]. Particulate organic carbon fluxes also increased with respect to fluxes recorded in the preceding winter, showing the same range of C/N ratios than those observed throughout the year (Figure S4A and S4B). These findings clearly illustrate that the intense cascade of shelf water is a quick way of providing large amounts of particulate organic matter to the northwestern Mediterranean deep basin, as it was previously suggested from hydrographical data [32].

**Figure 4:**
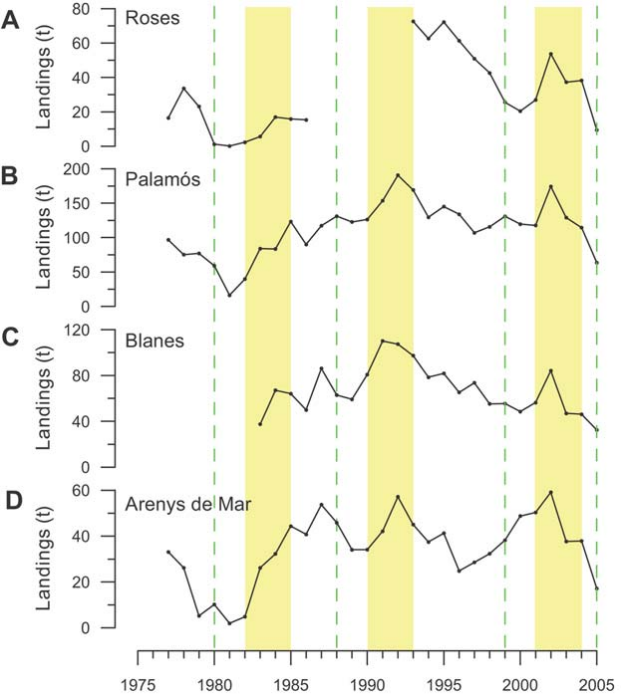
Temporal evolution of annual landings. (A–D) Available annual landings of *Aristeus antennatus* at the studied harbors since 1977. Green dashed lines indicate years when strong cascading events occurred. A similar temporal evolution of landings can be observed in all harbors, with an increasing trend and a peak of captures between 3 and 5 years after the cascading events (pale yellow bands).

Long-term analysis of landings of *Aristeus antennatus* shows a temporal correlation with intense cascading events (Figure 4A–4D), despite the variability inherent to these data caused by changes in fishing effort throughout the years and by the low accuracy of their acquisition. The temporal evolution of annual landings often shows relative minima during years affected by intense cascading and the following one, being the annual variability of all 4 annual landing time series significantly correlated at time-lag 0 (Figure S5; cross-correlation analysis using original data; correlation coefficients significant at 5% level). An increasing trend and a relative peak of landings occur from 3 to 5 years after cascading takes place (Figure 4A–4D). The mean landing values during the year directly affected by intense cascading, and the following one, were significantly lower when compared with mean landings between 3 and 5 years after these major events (Figure S6; Mann-Whitney *U* test, n = 50, p<0.001, using standardized values of each annual landing time series with the method of the percentage of the maximum). The same temporal evolution has been reported for landings in the fishing harbor of Palma de Mallorca (Figure S7) sited in the Balearic margin (see location in Figure 1), indicating that the dense shelf water cascading originating in the Gulf of Lions affects the populations of this deep-sea shrimp at a basin scale.

**Figure 5:**
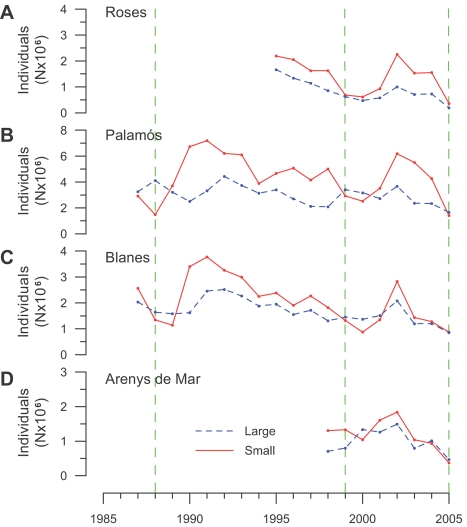
Temporal evolution in population structure. (A–D) Estimation of the abundance of small (continuous red line) and large (dotted blue line) individuals of *Aristeus antennatus* derived from the annual landings in the studied harbors. Green dashed lines indicate the years when major cascading events occurred. The temporal evolution of the population structure of *Aristeus antennatus* in all harbors was linked to those cascading events. The number of small individuals increased two and three years after the event, prior the increase in the number of large individuals, indicating that cascading events enhance the recruitment process of this species.

Subsequent increases in landings of *Aristeus antennatus* could be explained by an enhancement of recruitment of this species after major cascading events. The analysis of the temporal variability in the number of small and large individuals in landings can be used as a proxy to assess the population structure of *Aristeus antennatus* and additionally provide information on the inter-annual variability of the individual recruitment to fisheries. The historical data available suggest that during the years affected by cascading and the following one, the population of *Aristeus antennatus* has low numbers of both small and large individuals (Figure 5A–5D). However, two to three years after a major cascading, a large increase in the number of recruits (i.e. juveniles and young adults) to the fisheries occurs (Figure 5A–5D). Landings of large individuals, mainly comprised by an adult population, only start increasing 3 years after cascading, lasting for, at least, the following 2–3 years, which is the lifespan of this species (maximum age of 5 to 6 years) (Figure S1). Later, both large and small individuals show a decreasing trend, suggesting that fishing pressure may affects the populations of this species. This pressure could end up as overexploitation (see Figure S8). However, this does not occur probably because of the enhanced recruitment caused by the next cascading event (Figure 5A–5D).

How this enhanced recruitment exactly occurs remains unknown. For instance, the large transport of particulate organic matter to the deep basin during cascading events (Figure 2H and Figure S4A and S4B) might favor the nutritive condition of the adult population resulting in an improved reproductive output. Alternatively, the enhanced recruitment could be a direct consequence of a higher larval survival by increased food availability during the pelagic stages or during settlement to the bottom below 1000 m depth (see Introduction for a descriptive summary of *Aristeus antennatus* life cycle). Although the throphic argument seems the most reasonable explanation, decrease of predation pressure on larvae and juvenile individuals might also result on an enhanced recruitment to the fisheries. The turbidity anomaly generated in the Western Mediterranean Deep Water after cascading events (Figure 1) could avoid visual predation on larvae, or alternatively, predators of *Aristeus antennatus* could be also negatively affected by the dragging capacity of strong cascade currents.

Our findings provide a new understanding of the inter-annual landings fluctuations of *Aristeus antennatus*, which from the results shown in this study, appeared to be correlated to a climate-driven forcings. This knowledge will contribute to improve the management of this fishery, since the occurrence of major cascading events can be monitored and used as a predicting tool for administrating this living resource. Also, our results may provide an explanation to the paradox of a deep-sea fishery that after 70 years of heavy exploitation is still not depleted. However, climate models predict a reduction of dense water formation and cascading in the northwestern Mediterranean [32], [39]. Without these regenerative mechanisms, fishery pressure could quickly deplete the stocks of this valuable deep-sea living resource in this area. Deep water formation is also expected to decline in other areas of the world [40], in particular in high latitudes, where the largest demersal fisheries take place [16].

This study shows that a climate-driven phenomenon has a direct effect on deep-sea ecosystems, and consequently, on their living resources. The lateral input of energy to deep regions of the oceans caused by cascading can control biological processes at these depths. Applying our findings to a global fishery scenario, shelf water cascading sites identified worldwide [31], [41] could be considered as regions favorable for deep-sea demersal fisheries, in a similar way the upwelling zones are considered favorable regions for pelagic fisheries (Figure S9).

## Materials and Methods

The data presented in this study originate mainly from the analysis of historical landings of the deep-sea shrimp *Aristeus antennatus* (Risso 1816) from the fishing harbors in the Catalan margin (northwestern Mediterranean) that obtain the highest yields: Roses, Palamós, Blanes and Arenys de Mar (see Figure 1 for locations). These results are combined with data derived from instrumented moorings deployed in the northwestern Mediterranean continental slope and basin, which provided observations of water and sediment transport processes.

### Shrimp landings

Official landings of *Aristeus antennatus* were compiled from different sources. Annual landings time series were obtained directly from the statistical official archives of the fishermen associations of each port. Data were available since 1993, 1979, 1982, 1987 in Roses, Palamós, Blanes and Arenys de Mar, respectively. Additionally, annual landings from all these harbors, excluding Blanes, were published from 1977 until 1986 in the official statistical reports *Anuarios de Pesca Marítima* edited by **Dirección General de Pesca Marítima** (i.e. the Spanish governmental organism responsible of the Spanish fisheries until 1986). Both sources of information were combined to obtain historical records. Since 2001, daily landings are being recorded automatically by **Direcció General de Pesca i Afers Marítims** of the Autonomous Government of Catalonia. Daily landings previous to 2001 were available only from the statistical official archives of the fishermen associations of Palamós and Blanes. Cross-correlation analysis between the 6 harbor pairs obtained from the 4 annual landings time series were performed with the objective of identifying if temporal variability in landings were significantly correlated at the 4 studied harbors. Landing value standardization was done following the percentage of the maximum method, and a non-parametric Mann-Whitney *U* test was conducted in order to describe if average landings during specific years were statistically different than other.

### Population structure

Size categories of *Aristeus antennatus* in the statistical official archives of the fishermen associations were also available since 1996, 1987, 1987 and 1998 in Roses, Palamós, Blanes and Arenys de Mar, respectively. After these years, landings were separated by size and listed as two sales categories: small and large. The average weight of individuals per category was gathered directly from the landings of the studied harbors during winter-spring 2006. To estimate the population structure of the *Aristeus antennatus*, in terms of numbers of large and small specimens, captures from each category (in weight) were converted to number of individuals using 11 g and 29 g for small and large individuals, respectively (see Figure S1 for details). Fisheries of this species take places at depths from 500 down to 900 m, targeting individuals >21 mm CL. The large category includes mainly adult females over age 2 and a negligible proportion of adult males over age 4. The small category includes juvenile females over age 1, adult females under age 2.5 and adult males of age 2 to 3.

### Instrumented moorings

Two near-bottom mooring arrays were deployed in the northwestern Mediterranean continental slope and basin during 2004–2005. The mooring over the slope was located in the Cap de Creus Canyon at 500 m depth (red circle in Figure 1) and was instrumented with an Aanderaa RCM9 doppler current meter placed at 5 m above bottom and equipped with temperature, conductivity, pressure and turbidity sensors. The current meter sampling interval was set at 20 minutes. The mooring over the basin was deployed at 2350 m depth (green square in Figure 1) and was equipped with a Technicap PPS 5/2 conical sediment trap with a 1 m^2^ collecting area and 24 receiving cups. The trap was placed at 250 m above the bottom (i.e. at 2100 m depth) and the trap collecting intervals ranged from 5 to 15 days, depending on the season. Wave data from the study area were provided by **Puertos del Estado-Ministerio de Fomento** and were recorded by a Wavescan buoy deployed in 1200 m depth off Palamós (black triangle in Figure 1).

### Current meter data processing

Unfiltered raw data (speed and direction, temperature, conductivity, pressure and turbidity values) was checked for spurious data points and drifts. Temperature and conductivity data was fine tuned using contemporary CTD casts collected next to the mooring site. Turbidity data was converted into suspended sediment concentration using a general calibration curve for the northwestern Mediterranean [42].

### Sediment trap sample processing

Sediment trap cups were filled with a buffered 5% formaldehyde solution in 0.20 µm filtered seawater. After trap recovery, samples were stored at 4 °C until analysis. Trapped material was split with a WSD-10 wet sampler divider (McLane), filtered through pre-combusted pre-weighed Whatman GFF glass fiber filters and dried at 60° C for two days. Prior to C and N analysis, samples were weighed again in a Cahn microbalance to obtain dry weight. Particulate Carbon and Nitrogen (PC and PN) were analyzed with a LECO CN 2000 analyzer. Particulate Inorganic Carbon (PIC) was obtained by acid digestion (HCl 6M) of duplicate filters in a LECO CC 100 connected to the CN analyzer. POC was calculated from the difference between PC and PIC. C and N sample values were corrected with values obtained from filter blanks of sediment trap cup solution.

## Supporting Information

Figure S1 Schematics of the *Aristeus antennatus* growth. (A and B) Length-weight relationships (potential curves: W = a*CL^b^; W: weight; CL: carapace length) and modal size at each age (striped bars) of female (A) and male (B) individuals of *Aristeus antennatus*. Faded pink area represents juvenile individuals and pale blue area adult individuals. Fishable size classes are separated into small (red area on the length-weight curve) and large (blue area) sales categories used by the fishermen associations. The individual mean weight of each sales category was used to estimate the number of individuals on the landings (see Materials and Methods for details). Growth information was obtained from S1, S2, S3, S4, S5, S6, S7.
(1.86 MB EPS)

Figure S2 Temporal evolution of wave conditions. Significant wave height (H_0_) and wave peak period (T_p_) measured off Palamós in 1200 m water depth during 2004–2005 (see buoy location in Figure 1). Note that during the temporary fishery collapse associated with the winter 2005 major cascading event (see Figures 2 and 3) significant wave height was <3 m and wave peak periods <8 s, and only two storms (H_0_>4 m) that lasted for few days occurred. A similar wave regime was recorded in the preceding year during winter-time, and no unusual decrease in landings was observed.
(0.98 MB EPS)

Figure S3 Temporary fishery collapse in 1999. (A and B) Daily landings of *Aristeus antennatus* from October 1998 to October 1999 at Palamós (A) and Blanes (B). Note the temporary fishery collapse in March and April 1999, also co-occurred with the major dense shelf water cascading event originated in the Gulf of Lions in mid February 1999 (see Figure 6 in reference S8).
(1.00 MB EPS)

Figure S4 C/N ratio and POC flux in the basin. (A and B) Time series from March 2004 to April 2005 of the C/N ratio (A) and POC fluxes (B) of particles collected by the sediment trap deployed at 2100 m depth (250 m above the seafloor) in the northwestern Mediterranean basin. Note the abrupt increase of particle flux following the cascading event (see Figure 2H).
(0.99 MB EPS)

Figure S5 Cross-correlations between annual landing time series per each harbor pairs. (A–F) Cross-correlation coefficients per each of the 6 available harbor pairs. Note that the higher correlation coefficients for all pairs were obtained at time-lag 0, indicating that the temporal variability of the annual landings time series are correlated all over the fishing area. Dashed lines represent 5% significance level.
(1.08 MB EPS)

Figure S6 Annual mean landing values of years with major cascading compared to landing from years after cascading. Comparison of the annual mean landing values during cascading (i.e. landings registered at the year of the major cascading event, indicated as year 0, and the following one, indicated as year 1) with the annual mean landing values after cascading (i.e. landings registered between years 3, 4 and 5 after each major cascading occurred, the ones indicated in pale yellow bands in Figure 4). Annual mean landing values after cascading were significantly higher when compared with the annual mean values of the landings occurred during cascading (Mann-Whitney *U* test, n = 50, p<0.001, using standardized values of each annual landing time series with the method of the percentage of the maximum).
(0.70 MB EPS)

Figure S7 Temporal evolution of landings in the Balearic margin. Monthly time series of *Aristeus antennatus* landings from Palma de Mallorca harbor (see location in Figure 1) showing the same inter-annual variability observed in the fishing harbors considered in this study, a variability not understood until the new phenomena presented in this study. Landings from this fishing harbor come mainly from fishing boats operating in the northern side of Mallorca Island. Data after S4 and S9.
(0.93 MB EPS)

Figure S8 Decrease in landings during the inter-cascading period 1988–1999. A significant decrease in landings from year 1993 (i.e. year with higher landing values after the cascading occurred during winter 1988) up to year 1998 (i.e. one year before the cascade occurred during winter 1999) was found. The largest inter-cascading period available in our landing time series was used in order to correlate if fishery overexploitation could be related with this landing decrease (using standardized values of each annual landing time series with the method of the percentage of the maximum; linear regression equation parameters were: *a* = 1.2195, *b* = −0.0677, r = 0.7935, n = 27, regression slope *b* is significantly different from zero at p<0.001).
(4.57 MB EPS)

Figure S9 Worldwide dense shelf water cascade sites vs. annual catches in major FAO statistical areas. Sites where dense water cascades have been identified around the world oceans (red dots) [27], [35] compared with annual catches in the major FAO fishing areas for year 2002 (i.e. the most recent ones compiled in the latest FAO review of the state of world marine fishery resources, S10). Pelagic fishes include ISSCAAP groups 35, 36 and 37, demersal fishes: groups 31, 32 and 34; and crustaceans: groups: 42, 43, 44 and 45. Note how pelagic fisheries are more abundant in major upwelling areas (e.g. west coast of South America) and in contrast, the proportion of demersal fisheries is higher in major cascading areas (e.g. northern European margins).
(1.08 MB PDF)
