# Supplementary material for: Climate Influence on Deep Sea Populations
Source: PLoS One. 2008 Jan 16;3(1):e1431. doi: 10.1371/journal.pone.0001431 (PMC2174526; doi:10.1371/journal.pone.0001431)
Supplement: Figure S9 — Worldwide dense shelf water cascade sites vs. annual catches in major FAO statistical areas. Sites where dense water cascades have been identified around the world oceans (red dots) [27], [35] compared with annual catches in the major FAO fishing areas for year 2002 (i.e. the most recent ones compiled in the latest FAO review of the state of world marine fishery resources, S10). Pelagic fishes include ISSCAAP groups 35, 36 and 37, demersal fishes: groups 31, 32 and 34; and crustaceans: groups: 42, 43, 44 and 45. Note how pelagic fisheries are more abundant in major upwelling areas (e.g. west coast of South America) and in contrast, the proportion of demersal fisheries is higher in major cascading areas (e.g. northern European margins). (1.08 MB PDF) [file pone.0001431.s009.pdf]

Figure S9

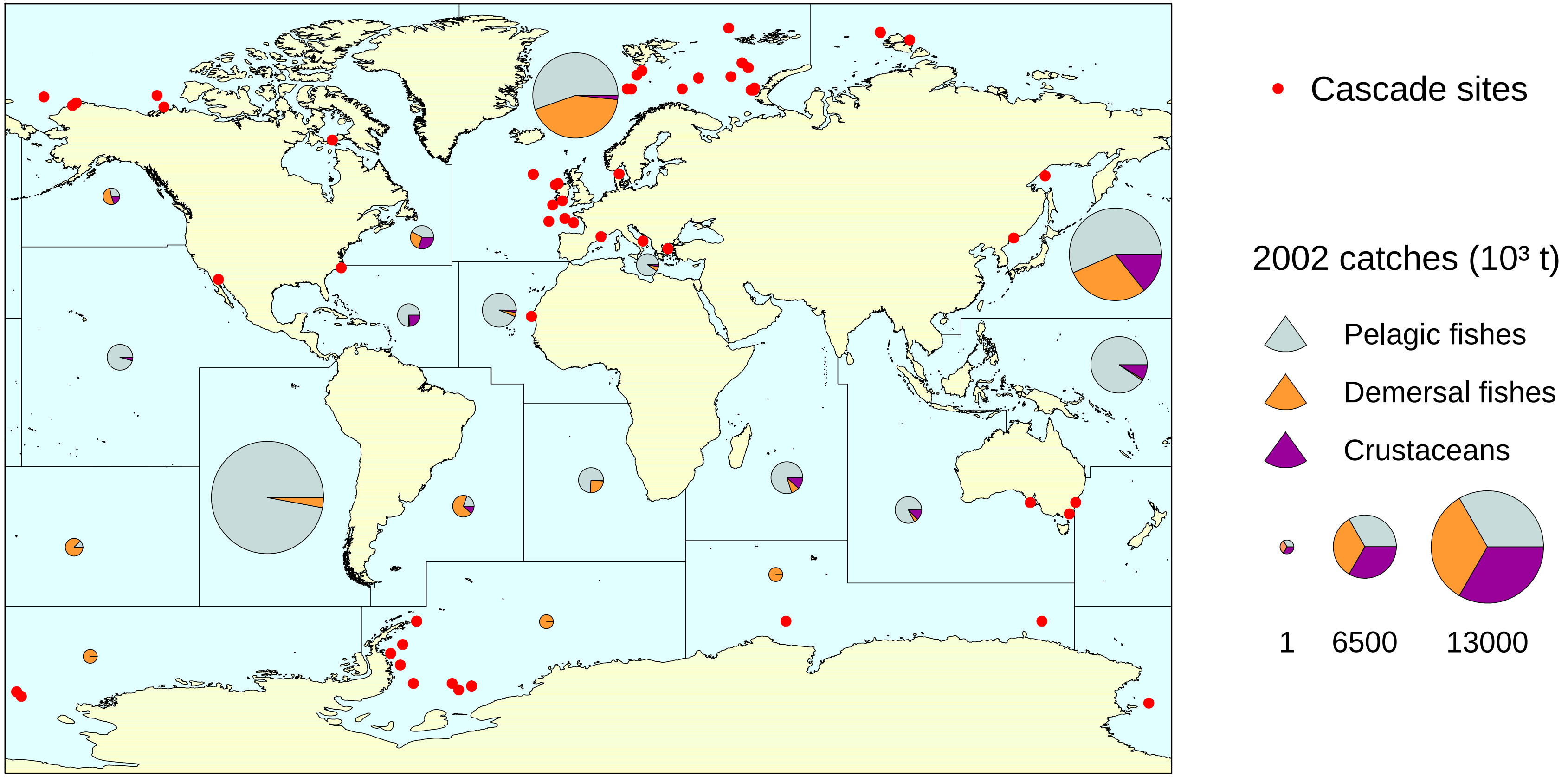
Cascade sites
2002 catches (10³ t)
Pelagic fishes
Demersal fishes
Crustaceans
1
6500
13000

Supporting references

S1. Sardà F, Demestre M (1987) Estudio biológico de la gamba *Aristeus antennatus* (Risso, 1816) en el Mar Catalán (NE de España). Inv Pesq 51: 213-232.

S2. Demestre, M (1990) Biología de la gamba rosada, *Aristeus antennatus* (Risso, 1816). PhD Dissertation, Univertitat de Barcelona.

S3. D’Onghia G, Capezzuto F, Mytilineou Ch, Maiorano P, Kapiris K, et al. (2005) Comparison of the population structure and dynamcs of *Aristeus antennatus* (Risso, 1816) between exploited and unexploited areas in the Mediterranean Sea. Fish Res 76: 22-38.

S4. Carbonell A (2005) Evaluación de la gamba rosada, *Aristeus antennatus* (Risso, 1816), en el Mar Balear. PhD Dissertation, Universitat de les Illes Balears.

S5. Ribeiro Cascalho A, Arrobas I (1982) *Aristeus antennatus* (Risso, 1816): some considerations about its biology and fishery in Portuguese waters. ICES C.M. K:6.

S6. Martinez Baño P, Vizuete F, Mas J (1988) Aspectos biológicos de la gamba roja *Aristeus antennatus* (Risso, 1816) a partir de las pesquerías del S.E. de la Península Ibérica. Bentos 6: 235-243.

S7. Lleonart J (1990) La pesquería de Cataluña y Valencia, descripción global y planteamiento de las bases para su seguimiento. Informe final CEE. Dirección General XIV-B-1 (1990).J. Lleonart, “La pesquería de Cataluña y Valencia, descripción global y planteamiento de las bases para su seguimiento” (Tech. Rep. CEE. XIV-B-1, 1990).

S8. Béthoux JP, Durrieu de Madron X, Nyffeler F, Tailliez D (2002) Deep water in the western Mediterranean: peculiar 1999 and 2000 characteristics, shelf formation hypothesis, variability since 1970 and geochemical inferences. J Mar Sys 33–34: 117–13.

S9. Carbonell A, Carbonell M, Demestre M, Grau A, Montserrat S (1999) The red shrimp *Aristeus antennatus* (Risso, 1816) fishery and biology in the Balearic Islands, Western Mediterranean. Fish Res 44: 1-13.

S10. FAO (2005) Review of the state of world marine fishery resources. FAO Fisheries Technical Paper No. 457. Rome, FAO. 235 p.
